# Digital literacy in the university setting: A literature review of empirical studies between 2010 and 2021

**DOI:** 10.3389/fpsyg.2022.896800

**Published:** 2022-09-06

**Authors:** Nieves Gutiérrez-Ángel, Jesús-Nicasio Sánchez-García, Isabel Mercader-Rubio, Judit García-Martín, Sonia Brito-Costa

**Affiliations:** ^1^Departamento de Psicología, Área de Psicología Evolutiva y de la Educación, Universidad de Almería, Almeria, Spain; ^2^Departamento de Psicología, Sociología y Filosofía, Universidad de León, Leon, Spain; ^3^Departamento de Psicología Evolutiva y de la Educación, Universidad de Salamanca, Salamanca, Spain; ^4^Instituto Politécnico de Coímbra, Coimbra, Portugal; ^5^Coimbra Education School, Research Group in Social and Human Sciences Núcleo de Investigação em Ciências Sociais e Humanas da ESEC (NICSH), Coimbra, Portugal

**Keywords:** digital literacy, pre-service & teacher education, higher education, teachers', transversal competences

## Abstract

The impact of digital devices and the Internet has generated various changes at social, political, and economic levels, the repercussion of which is a great challenge characterized by the changing and globalized nature of today's society. This demands the development of new skills and new learning models in relation to information and communication technologies. Universities must respond to these social demands in the training of their future professionals. This paper aims to analyze the empirical evidence provided by international studies in the last eleven years, related to the digital literacy of university students, including those pursuing degrees related to the field of education. Our findings highlight the fact that the digital literacy that is offered in universities to graduate/postgraduate students, in addition to treating digital literacy as a central theme, also focuses on perceived and developed self-efficacy. This is done by strengthening competencies related to digital writing and reading, the use of databases, the digital design of content and materials, and the skills to edit, publish or share them on the web, or applications aimed at treating digital literacy as emerging pedagogies and educational innovation. Secondly, we found studies related to digital competencies and use of the Internet, social networks, web 2.0, or the treatment of digital risks and their relationship with digital literacy. Thirdly, we found works that, in addition to focusing on digital literacy, also focused on different psychological constructs such as motivation, commitment, attitudes, or satisfaction.

**Systematic review registration:** https://www.scopus.com/home.uri; https://www.recursoscientificos.fecyt.es/.

## Introduction

The concept of digital literacy (DL) appears for the first time in the works of Zurkowski (1974), for whom it is an ability to identify, locate, and examine information. However, despite its novelty, the conceptions it encompasses have been changing (Lim and Newby, 2021). Proof of this are the contributions of Gilster (1997) who combines the idea that DL is also closely linked to skills such as access, evaluation, and management of information used in learning processes. Digital learning is understood as the set of technical-procedural, cognitive, and socio-emotional skills necessary to live, learn, and work in a digital society (Eshet-Alkalai, 2012; European Commission, 2018). It is related to reading, writing, calculation skills, and effective use of technology in personal, social, and professional areas. It is also considered inseparable from the social and educational needs of the society in which we live (Larraz, 2013; Brata et al., 2022). Therefore, we refer to a concept that has several aspects including the technological aspect, the informative and multimedia aspect, and the communicative aspect. It involves a complete process and multiple literacies (Gisbert and Esteve, 2011; Lázaro, 2015; Valverde et al., 2022). It requires mastery of certain competencies related to the identification of training needs, access to information in digital environments, the use of ICT tools to manage information, interpretation, and representation of information, and the evaluation of information and the transmission of information (Covello and Lei, 2010; Walsh et al., 2022).

### Digital literacy in university students

In recent years, society has undergone enormous changes with the digitalization of many of its spheres at the information level, the communication level, the level of knowledge acquisition, the level of the establishment of social relations, and even the level of leisure. Thus, our habits and means of accessing, managing, and transforming information have also changed (European Union, 2013; Cantabrana and Cervera, 2015; Allen et al., 2020; López-Meneses et al., 2020).

These developments have also had a great impact on the educational field, in which we have to rethink firstly what kind of students we are training in terms of the skills they need in today's society, and secondly, whether we are training a profile of future teachers capable of training a student body that uses information and communication technologies as something inherent to their own personal and social development. In short, digital communication has changed practices related to literacy and has gained great relevance in the development of knowledge in the twenty-first century (Comisión Europea, 2012, 2013; European Commission, 2012; OECD, 2012; Unión Europea, 2013; Instituto Nacional de Tecnologías Educativas y Formación del Profesorado, 2017; Gudmundsdottir and Hatlevik, 2018; Pérez and Nagata, 2019; Fernández-de-la-Iglesia et al., 2020).

The European Commission (2013) indicates that initial teacher training (IDT) should integrate teachers' digital literacy, betting on the pedagogical use of digital tools, enabling them to use them in an effective, appropriate, and contextualized manner. This teaching competence should be characterized by having a holistic, contextualized, performance-, function-, and development-oriented character. In short, it is about incorporating and adequately using ICT as a didactic resource (Cantabrana and Cervera, 2015; Castañeda et al., 2018; Tourón et al., 2018; Chow and Wong, 2020; Vodá et al., 2022).

In this sense, according to the work of Krumsvik (2009), the CDD (*competencia digital docente de los profesores*–digital competency training for teachers) is composed of four components: basic digital skills (Bawden, 2008), didactic competence with ICT (Koehler and Mishra, 2008; Gisbert and Esteve, 2011), learning strategies, and digital training or training.

While at the Spanish level, the Common Framework of Digital Teaching Competence of the National Institute of Educational Technologies and Teacher Training (INTEF, 2017) standardizes it in five areas: information and information literacy, communication and collaboration, digital content creation, security, and problem solving (López-Meneses et al., 2020). Recently, they have been consolidated as competencies that must be acquired by any university student, along with the knowledge, skills, and attitude that make up a digitally competent citizen (Recio et al., 2020; Indah et al., 2022).

### Digital literacy in future teachers

Several efforts have been made to equip future teachers with these competencies through different standards and frameworks to the level of learning acquired (Fraser et al., 2013; INTEF, 2017; UNESCO, 2018). However, how to work these competencies in initial training is still a hotly debated topic, in which special attention is paid to the promotion of experiences of a pedagogical and innovative nature to transform teaching practices, involving the integration of technologies in the classroom, as stated in the Horizon Report 2019 for the Higher Education (Educause, 2019; Le et al., 2022).

Universities are in a moment of transformation, from a teacher-focused teaching model to a model based on active learning through the use of digital technologies, giving rise to a new type of education in which the use of digital devices is intrinsic (Area, 2018; Aarsand, 2019). If digital resources and devices are an inescapable part of current and future teaching practice, digital competency training for future teachers becomes extremely relevant, given that teachers need to acquire these competencies in their initial training to integrate them into their practices as future teachers. That is, the digital competence (DC) acquired during their initial training significantly predicts the integration of technologies in future teaching practice (Nikou and Aavakare, 2021), which could range from basic digital literacy to the integration of technologies in their daily teaching practice (Gisbert et al., 2016; Alanoglu et al., 2022). Several studies have defined the different indicators that make up DC (Siddiq et al., 2017; González et al., 2018; Rodríguez-García et al., 2019; Cabero-Almenara and Palacios-Rodríguez, 2020).

This calls for a new paradigm, in which future teachers must be digitally literate, in terms of the application of active methodologies, digital competencies, and the use of innovative strategies, styles, and approaches (Garcia-Martin and Garcia-Sanchez, 2017; Gómez-García et al., 2021).

Currently, literacy workshops for future professionals are being carried out in a timely and precise manner from customized short training capsules to specific semester-long subjects in undergraduate or postgraduate studies. The training is focused on several specific aspects of digital literacy, but there is a lack of experience in imparting comprehensive digital training. In addition, there are just a few interactions with professional experts in such literacy (Ata and Yildirim, 2019; Campbell and Kapp, 2020; Domingo-Coscolla et al., 2020; Tomczyk et al., 2020; Vinokurova et al., 2021).

### The present study

For the present study, we based our approach on quality and current education, in which DC was postulated as a key element for the development of students. The educational system was tasked with preparing them for their full development and participation in society (OECD, 2011). For this reason, digital literacy is understood as an essential requirement for development in the society in which we live, based on the promotion of strategies related to searching, obtaining, processing, and communicating information. All these aspects have been consolidated as the dimensions of literacy in the twenty-first century (Piscitelli, 2009; Martín and Tyner, 2012). It is, therefore, necessary to understand the reality of this subject and to investigate how these practices are being developed in the context of work. And secondly, it is equally necessary to implement new interventions and lines of research that respond to this urgent need for literacy required by today's society. Therefore, we posed the following research questions: What psychoeducational and learning variables are key in digital literacy? What is the current situation internationally regarding digital literacy in all disciplines in pre-service teacher education? What are the differences in digital literacy requirements pre and post pandemic?

#### Objective

The objective of this study is to analyze the empirical evidence provided by international studies from 2010 to 2021 related to the digital literacy of university students, including those who are pursuing careers related to the educational field.

Relevant differences will be observed in the contributions in empirical evidence from international studies pre-post-pandemic; and drawn from diverse cultural backgrounds (Spanish-Latin, Portuguese, Finnish, etc.,), gender, and personal digital resources.

## Materials and methods

The systematic review is composed of four phases, following the model of Miller et al. (2016) and Scott et al. (2018).

**Figure 1:**
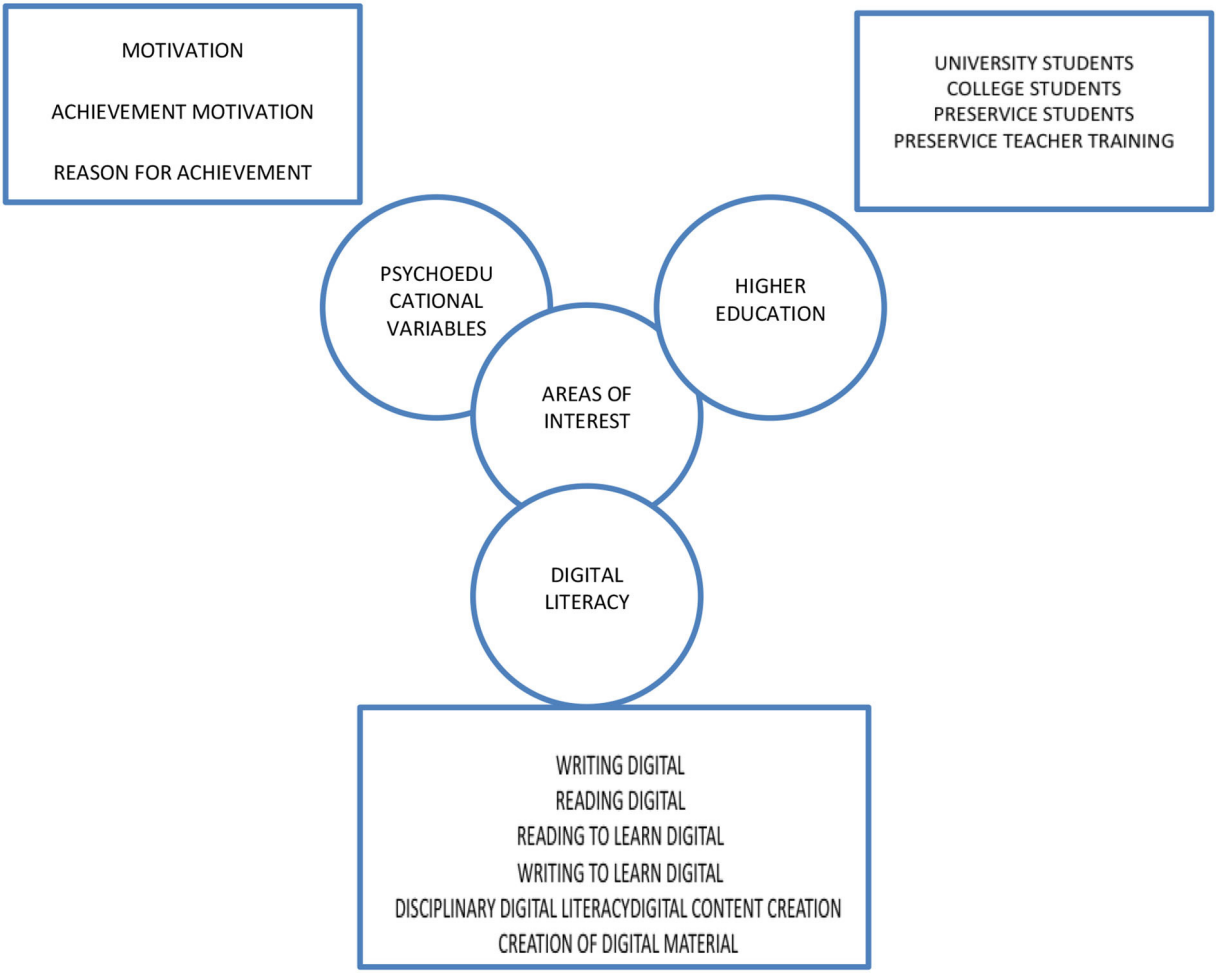
Diagram of search terms used in the systematic review.

PHASE 1: Search terms: In this phase, we developed a schematic of search terms from Web of Science and Scopus databases. We also accessed the databases to locate specific studies that were referenced in the publications that we found in the databases during our initial search. The schematic of terms and thematic axes that were used as a starting point for scanning both databases for anything related to the descriptor “digital” and the descriptor “literacy” is presented in Figure 1.

PHASE 2: Selection process based on inclusion and exclusion criteria. The following selection criteria were applied: year of publication between 2010 and 2021, availability of full text, and language of publication in English, Portuguese, or Spanish. Once the first results were obtained, they were selected based on title, abstract, and the use of standardized instruments in their methodology. We rejected the studies that used “*ad hoc*” instruments to measure digital competence.

In addition, the selection indicators provided by Cooper and Hedges (1994) and Cooper (2009) were used, such as peer-reviewed journals, referenced databases, and citation indexes.

PHASE 3: Analysis of methodological quality and indicators based on scientific evidence. Following Torgerson (2007) and Risko et al. (2008) and taking into consideration the MQQn (Risko et al., 2008), we used seven indicators to analyze the quality and effectiveness of the studies (Acosta and Garza, 2011). These were: alignment of theory, findings, reliability and validity, descriptive details of participants and the study, sample, and consistency of findings and conclusions with the data (Risko et al., 2008). Alternatively, evidence-based indicators were also used along with study effect sizes (Díaz and García, 2016; Canedo-García et al., 2017).

PHASE 4: Reliability and outcomes. Reliability was established for both the selection criteria and the coding criteria during each phase, to evidence the replicability of the results. In addition, the results entailed a qualitative analysis of the selected studies, the central arguments, and the evidence provided in a modulated way to address the research questions.

**Figure 2:**
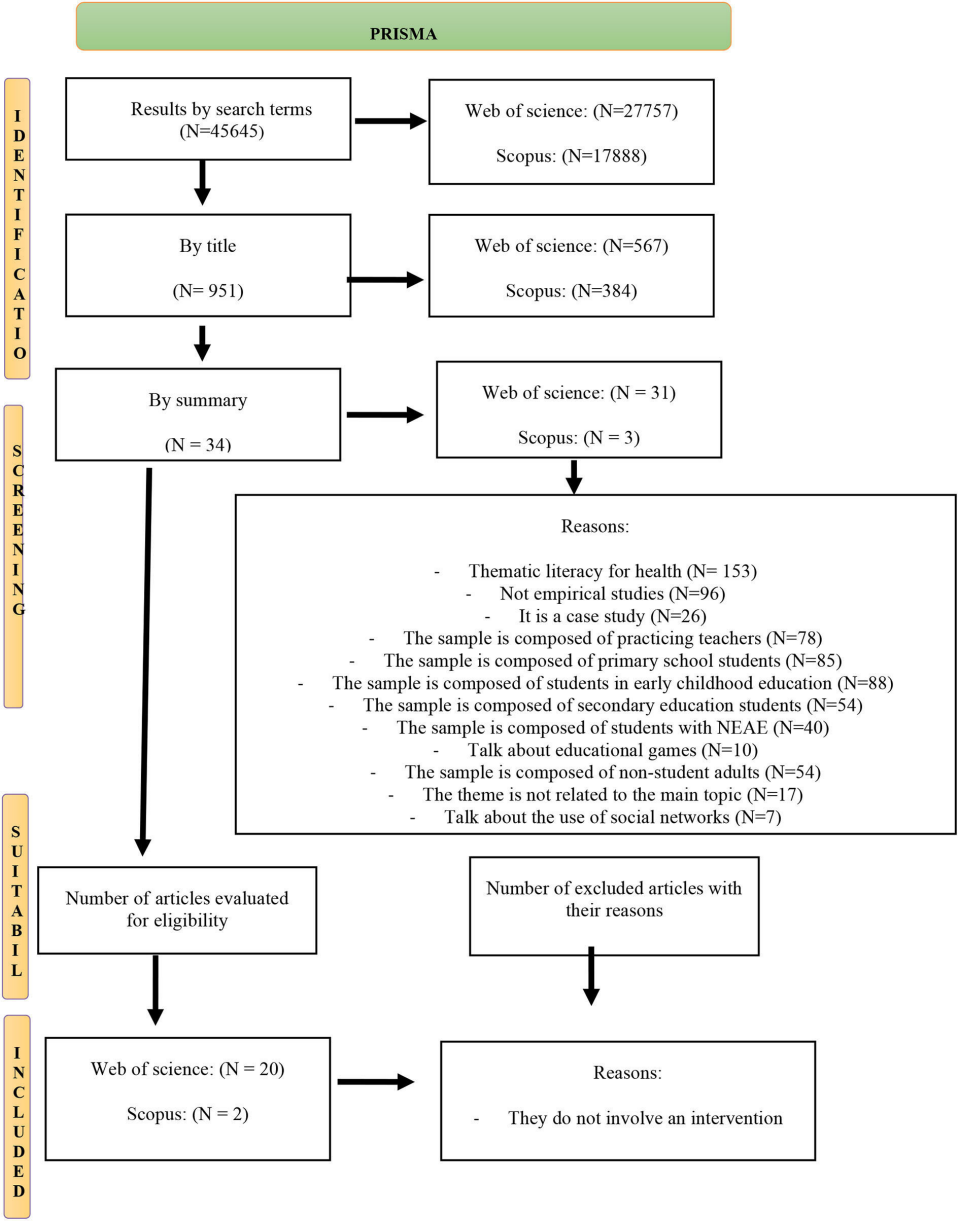
Flowchart of search results of empirical studies in databases applying the criteria of Moher et al. (2009) and Page et al. (2021).

Therefore, the procedure to be followed was documented and charted according to the PRISMA statement (Moher et al., 2009; Page et al., 2021) (see Figure 2). Likewise, an analysis was undertaken of the key foci in the various studies to highlight the relevant findings and evidence they provided in this regard. The key focus of our work was: first, to analyze the documents related to the digital literacy of university students; second, to identify which variables affect digital literacy; and third, to undertake a comparative analysis between the different variables that were analyzed.

## Results

All the selected studies had as samples university students who were pursuing some type of degree or postgraduate degree related to education, and therefore, studying to become future teachers. An intervention design was presented that corresponds to a pre-intervention, the intervention itself, and a post-intervention using techniques such as the activation of prior knowledge, instructions, emulation, and subsequent tests. We also found studies that had an experimental design assessing control groups and experimental groups (Kajee and Balfour, 2011; Kuhn, 2017; Pequeño et al., 2017; Sharp, 2018; Lerdpornkulrat et al., 2019).

In the case of those responsible for the intervention, practically in all cases, the teacher acts as such, with one or two of them taking the lead. Although the presence of specialized personnel should also be highlighted, as is the case of the work elaborated by Alfonzo and Batson (2014) and Elliott et al. (2018) in which a professional librarian also intervened. Or, in the work detailed by Ball (2019), where a consultant who is not a teacher but a professional expert in the use of digital devices and trained for such an occasion by a responsible brand (Apple) carried out the training at the center.

If we examine the constructs or competencies covered by the works selected in our search, we find that all of them, in addition to dealing with digital literacy, also focus on self-efficacy perceived and developed through digital literacy.

The results of our study could be understood under different themes.

First, we found studies that referred to digital competence and other educational issues. Within them, we found a series of competencies that are emphasized such as digital writing and reading. Research developed from digital media, such as databases, web, or applications aimed at the treatment of digital literacy was noted as emerging pedagogies and educational innovation. The digital design of content and materials and the skills to edit, publish or share them, and competencies related to mathematics and its digital literacy, formed part of digital literacy.

Second, we found studies related to digital competence and the use and employment of the Internet, social networks, web 2.0, and the treatment of digital risks and their relationship with digital literacy.

Third, we found works that in addition to focusing on digital literacy, also focused on different psychological constructs such as motivation, commitment, attitudes, or satisfaction (Tables 1, 2).

**Table 1:** Summary of the results found.

**Research**	**Participants**					**Construct and competence**	**Instructional procedure**	**Instructional techniques**	**Instructional strategies**
	**Sample**	**Groups**	**Design**	**Sampling and inclusion and exclusion criteria**	**Teachers**				
Alfonzo and Batson (2014)	*N* = 20 university doctoral students (future teachers)	Do not specify	Pre-post intervention	Intentional sampling	N Teachers = 2. A teacher and a librarian	Digital literacy/ digital research/research software/ sdigital databases/self-efficacy	Digital search—apa standards—applications Resource management	Activation of previous knowledge-scaffolding Self-instructions Collaborative/ individual emulation Visualization	Specific grants Colloquium Planning-Reinforcement Review Selection
Ata and Yildirim (2019)	*N* = 295 university students (future teachers)	Do not specify	Pre-post intervention	Intentional sampling	N Teachers = 1	Digital literacy/internet/ social media/perception/ digital reading/digital writing/self-efficacy	Training course	Activation of previous knowledge-scaffolding Self-instructions Collaborative/ individual emulation Visualization	Colloquium Planning-Reinforcement Review Selection
Ball (2019)	Do not specify	Do not specify	Pre-post intervention	Do not specify	Specialized personnel	Digital literacy/digital writing/digital material/creation/editing// media literacy/cybersecurity/ self-efficacy	BA Writing and Publishing Program. emphasis on writing, researching, evaluating and reviewing articles in a digital environment	Activation of previous knowledge-scaffolding Self-instructions Collaborative/individual emulation Visualization	Colloquium Planning-Reinforcement Review Selection
Botturi (2019)	*N* = 26 university students (future teachers)	Do not specify	Pre-post intervention	Intentional sampling	N Teachers = 1	Digital literacy/access to information/digital content creation/content sharing/self-efficacy	Specific face-to-face program of 2 credits DML education course with 12 2-h sessions	Activation of previous knowledge-scaffolding Self-instructions Collaborative/individual emulation Visualization	Colloquium Planning-Reinforcement Review Selection
Campbell and Kapp (2020)	*N* = 4 university students (future teachers)	Do not specify	Pre-post intervention	Do not specify	N Teachers = 1	Digital literacy/self-efficacy/motivation	Training course Graduate Certificate in Education (PGCE)	Activation of previous knowledge-scaffolding Self-instructions Collaborative/individual emulation Visualization	Colloquium Planning-Reinforcement Review Selection
Carl and Strydom (2017)	*N* = 11 university students (future teachers)	Do not specify	Pre-post intervention	Intentional sampling	*N Teachers = 1*	Digital literacy/E-portfolio/self-efficacy/motivation	Digital content design—digital material design	Activation of previous knowledge-scaffolding Self-instructions Collaborative/individual emulation Visualization	Colloquium Planning-Reinforcement Review Selection
Domingo-Coscolla et al. (2020)	*N* = 11 university students (future teachers)	Do not specify	Pre-post intervention	Intentional sampling	N Teachers = 11	Digital literacy/diversity/innovation/self-efficacy/motivation	FIMTD project	Activation of previous knowledge-scaffolding Self-instructions Collaborative/individual emulation Visualization	Colloquium Planning-Reinforcement Review Selection
Elliott et al. (2018)	*N* = 48 university students (future teachers)	Do not specify	Pre-post intervention	Intentional sampling	Support staff—library staff	Digital literacy/digital writing/digital material/self-efficacy	Module focused on theories of learning and development—sociological module focused on educational inequalities	Activation of previous knowledge-scaffolding Self-instructions Collaborative/individual emulation Visualization	Colloquium Planning-Reinforcement Review Selection
Elphick (2018)	*N* = 949 university students (future teachers)	Do not specify	Pre-post intervention	Intentional sampling	N Teachers = 1	Digital literacy/attitude/motivation/ self-efficacy	Use of iPad in education and on a day-to-day basis	Activation of previous knowledge-scaffolding Self-instructions Collaborative/individual emulation Visualization	Colloquium Planning-Reinforcement Review Selection
Gabriele et al. (2019)	*N* = 141 university students (future teachers)	Do not specify	Pre-post intervention	Intentional sampling	Do not specify	Digital literacy/attitude/web 2.0/gamification/self-efficacy	Training course	Activation of previous knowledge-scaffolding Self-instructions Collaborative/individual emulation Visualization	Colloquium Planning-Reinforcement Review Selection
Gill et al. (2015)	*N* = 11 university students (future teachers)	Do not specify	Pre-post intervention	Intentional sampling	Do not specify	Digital literacy/pre-preparation/digital knowledge/self-efficacy	Application of practical knowledge from different subjects of the career	Activation of previous knowledge-scaffolding Self-instructions Collaborative/individual emulation Visualization	Specific grants Colloquium Planning-Reinforcement Review Selection
Hamutoglu et al. (2019)	*N* = 47 university students (future teachers)	Do not specify	Pre-post intervention	Intentional sampling	N Teachers = 1	Digital literacy/attitude/digital learning/self-efficacy/motivation	Training course once a week for 3 h per week	Activation of previous knowledge-scaffolding Self-instructions Collaborative/individual emulation Visualization	Colloquium Planning-Reinforcement Review Selection
Istenic et al. (2016)	*N* = 115 university students (future teachers)	Do not specify	Pre-post intervention	Intentional sampling	Do not specify	Digital literacy/digital content design/digital mathematics/self-efficacy	Creation of digital stories—design of digital content—design of digital materials	Activation of previous knowledge-scaffolding Self-instructions Collaborative/individual emulation Visualization	Specific grants Colloquium Planning-Reinforcement Review Selection
Kajee and Balfour (2011)	*N* = 20 university students (future teachers)	GE = 10 GC = 10	Pre-post intervention	Intentional sampling	N Teachers = 1	Academic Literacy/Digital Writing/Digital Research/Self-Efficacy	Self-instructional/online classes in specific labs	Activation of previous knowledge-scaffolding Self-instructions Collaborative/individual emulation Visualization	Specific grants Colloquium Planning-Reinforcement Review Selection
Kuhn (2017)	*N* = 20 university students (future teachers)	GE = 12 GE2 = 5 GC = 3	Pre-post intervention	Intentional sampling	Do not specify	Digital literacy/attitude/digital skills/motivation/autonomy/ self-efficacy	Digital Practice and PLE	Activation of previous knowledge-scaffolding Self-instructions Collaborative/individual emulation Visualization	Colloquium Planning-Reinforcement Review Selection
Lerdpornkulrat et al. (2019)	*N* = 584 university students (future teachers)	GE = 321 GC = 263	Pre-post intervention	Intentional sampling	N Teachers = 1	Digital literacy/motivation/self-efficacy	Training course	Activation of previous knowledge-scaffolding Self-instructions Collaborative/individual emulation Visualization	Colloquium Planning-Reinforcement Review Selection
Paige et al. (2016)	*N* = 31 university students (future teachers)	Do not specify	Pre-post intervention	Intentional sampling	Do not specify	Digital literacy/digital content design/digital mathematics	Creation of digital stories—design of digital content—design of digital materials	Activation of previous knowledge-scaffolding Self-instructions Collaborative/individual emulation Visualization	Slowmation—digital narratives—round tables—interviews—oral evaluations
Pequeño et al. (2017)	*N* = 54 university students (future teachers)	GE = 31 GC = 24	Pre-post intervention	Intentional sampling	Do not specify	Digital literacy/digital narrative/self-efficacy	Application of practical knowledge from different subjects of the career	Activation of previous knowledge-Scaffolding Self-instructions Collaborative/individual emulation Visualization	Colloquium Planning-Reinforcement Review Selection
Robertson et al. (2012)	*N* = 150 university students (future teachers)	Do not specify	Pre-post intervention	Intentional sampling	N Teachers = 2	Digital literacy/new pedagogies/multiliteracy/self-efficacy	Creation of digital stories—thoughtful writing	Activation of previous knowledge-scaffolding Self-instructions Collaborative/individual emulation Visualization	Specific aid Colloquium Planning-Reinforcement Review Selection -Sharing
Sharp (2018)	*N* = 51 university students (future teachers)	GE = 20 GE2 = 20 GC = 11	Pre-post intervention	Intentional sampling	Do not specify	Digital literacy/attitude/digital skills/motivation/autonomy/ self-efficacy	Creation of a blog, —asynchronous discussion, —wiki, —microblog	Activation of previous knowledge-scaffolding Self-instructions Collaborative/individual emulation Visualization	Colloquium Planning-Reinforcement Review Selection
Tomczyk et al. (2020)	*N* = 227 university students (future teachers)	Do not specify	Pre-post intervention	Intentional sampling	Do not specify	Digital literacy/digital inclusion/digital risks/digital content/self-efficacy	SELI Platform	Activation of previous knowledge-scaffolding Self-instructions Collaborative/individual emulation Visualization	Colloquium Planning-Reinforcement Review Selection
Vinokurova et al. (2021)	Do not specify	Do not specify	Do not specify	Do not specify	Do not specify	Digital literacy/self-efficacy	Training course	Activation of previous knowledge-scaffolding Self-instructions Collaborative/individual emulation Visualization	Colloquium Planning-Reinforcement Review Selection

**Table 2:** Summary of the interventions found.

**Research**	**Materials**	**Instructor role**	**Student role**	**Student grouping**	**Implementation/ Context**	**Program duration**	**Intervention results**	**Comments**
Alfonzo and Batson (2014)	Texts/documents—specific computer applications—material with indications	Teacher—Researcher	Developer of each activity	Small group	Researcher/virtual	For 4 days	Greater use of digital tools than before training	Has a sparse sample
Ata and Yildirim (2019)	Does not specify	Teacher—Researcher	Developer of each activity	Great group	Researcher/face-to-face	An academic year	Increasing digital competence	It should apply more evaluation tools
Ball (2019)	Dashboard—training modules—Wikipedia guidelines and rules	Teacher—Researcher	Developer of each activity	Small group	Researcher/face-to-face	An academic year	Increasing digital competence	Does not indicate the method
Botturi (2019)	Texts/documents—specific computer applications—material with indications	Teacher—Researcher	Developer of each activity	Great group	Researcher	4 months	Increasing digital competence	Has a sparse sample
Campbell and Kapp (2020)	Texts/documents—specific computer applications—material with indications	Teacher—Researcher	Developer of each activity	Great group	Researcher/virtual	5 months	Increasing digital competence	Has a sparse sample
Carl and Strydom (2017)	Texts/documents—specific computer applications—material with indications	Teacher—Researcher	Developer of each activity	Small group	Researcher/virtual	Do not specify	Great interest and motivation on the part of the participants	Does not use standardized instruments
Domingo-Coscolla et al. (2020)	Texts/documents—specific computer applications—material with indications	Teacher—Researcher	Developer of each activity	Great group	Researcher	Do not specify	Increasing digital competence	Has a sparse sample/does not indicate duration
Elliott et al. (2018)	Weekly Lectures-seminars-online resources-library	Teacher—Researcher	Developer of each activity	Small group	Researcher/face-to-face	An academic year	Increased digital expertise and dominance	Has a sparse sample
Elphick (2018)	Conferences and seminars	Teacher—Researcher	Developer of each activity	Great group	Researcher/face-to-face	One semester	Increased digital expertise and dominance	Does not use standardized instruments
Gabriele et al. (2019)	Power point presentations—introductory videos of the software-brochures—applications created *ad hoc*	Teacher—Researcher	Developer of each activity	Great group	Researcher/face-to-face	10 months	Increasing digital competence	Has a sparse sample
Gill et al. (2015)	Texts/documents—specific computer applications—material with indications	Teacher—Researcher	Developer of each activity	Small group	Researcher/virtual	For 3 years	Practical knowledge of the application of ICT as a learning tool	Has a sparse sample
Hamutoglu et al. (2019)	Texts/documents—EDMODO	Teacher—Researcher	Developer of each activity	Great group	Researcher/face-to-face	5 weeks	Increasing digital competence	Has a sparse sample
Istenic et al. (2016)	Texts/documents—specific computer applications—material with indications	Teacher—Researcher	Developer of each activity	Small group	Researcher/virtual	An educational technology course in the academic year 2011–2012	Creation of digital content for the teaching of mathematics	Does not use standardized instruments
Kajee and Balfour (2011)	Texts/documents—computer applications-Laboratory with computers–standalone server—printer	Teacher—Researcher through 40 workstations	Developer of each activity through 40 workstations	Small group/face-to-face	Researcher Specific laboratory	Two semesters of 14 weeks duration	GE improvements greater than GC	Has a sparse sample
Kuhn (2017)	Texts/documents—specific computer applications—material with indications	Teacher—Researcher	Developer of each activity	Small group	Researcher/virtual	An academic year	GE1 and GE2 improvements greater than GC	Has a sparse sample
Lerdpornkulrat et al. (2019)	Power point presentations—introductory videos of the software-brochures	Teacher—Researcher	Developer of each activity	Small group	Researcher/face-to-face	13 sessions	Increased self-efficacy in relation to standards and expectations	It should apply more evaluation tools
Paige et al. (2016)	Texts/documents—specific computer applications—material with indications	Teacher—Researcher	Developer of each activity	Small group	Researcher/virtual	Do not specify	Creation of digital content for the teaching of mathematics	Does not use standardized instruments
Pequeño et al. (2017)	Texts/documents—specific computer applications—material with indications	Teacher—Researcher	Developer of each activity	Small group	Researcher/virtual	An academic year	GE improvements greater than GC	Has a sparse sample
Robertson et al. (2012)	Texts/documents—computer applications—Photo Story 3 program	Teacher—Researcher	Developer of each activity	Small group	Researcher/virtual	For 3 years: 10 months	New learning and means of expression	Has a sparse sample
Sharp (2018)	Texts/documents—specific computer applications—material with indications	Teacher—Researcher	Developer of each activity	Small group	Researcher/face-to-face	Two semesters	GE1 and GE2 improvements greater than GC	Has a sparse sample
Tomczyk et al. (2020)	Texts/documents—SELI platform	Teacher—Researcher	Developer of each activity	Great group	Researcher/virtual	Do not specify	Increasing digital competence	Does not indicate the process
Vinokurova et al. (2021)	Texts/documents—specific computer applications—material with indications	Teacher—Researcher	Developer of each activity	Great group	Researcher/virtual	Do not specify	Increasing digital competence	Omits data for possible replicability

Regarding instructional literature, we found a large number of results on mass training programs or courses in which digital literacy was the focus. Examples include a course offered in which students could sign up to, or modules taught during the teaching of a subject. We also found investigations on interventions that had been carried out through different subjects in the study program from where the sample was taken. In this case, the samples were taken on an *ad hoc* basis from a specific student body which the researcher intentionally decided based on a previous intervention experience with them (Ata and Yildirim, 2019; Ball, 2019; Campbell and Kapp, 2020; Domingo-Coscolla et al., 2020; Tomczyk et al., 2020; Vinokurova et al., 2021).

In terms of material resources, all the studies used some type of documentation (digital or not) with instructions on the development of the activities, in which the students were provided with what to do and the steps to follow. In this case, the development scenario was both online and face-to-face, based on different activities given through workshops or seminars for their development.

It should also be noted that in those investigations in which the intervention itself required a specific application or program, the same was used, specifically, and even the intervention had a specific scenario since it was carried out in person in specialized laboratories where experts and specific material was available for this purpose. As an example of these specific materials, in our results, we found the use of the Photo Story 3, Dashboard, and Wikipedia, as well as the EMODO program or the SELI platform (Kajee and Balfour, 2011; Robertson et al., 2012; Ball, 2019; Hamutoglu et al., 2019; Tomczyk et al., 2020).

Regardless of the setting and the program or application employed, we can classify the duration of these interventions into two broad groups: those that had a duration of <1 semester, and those that had an intervention whose duration ranged from one semester to one academic year.

Regarding the instruments used, it should be noted that most of them used survey forms as an evaluation instrument, either by the researcher or by the students. In addition, it is usually used as a resource to collect information of a personal nature and about one's own experience throughout the intervention. We must also highlight the fact that in many of the results found, this form was used digitally or virtually, abandoning the old paper forms (Kajee and Balfour, 2011; Robertson et al., 2012; Carl and Strydom, 2017; Elliott et al., 2018; Ball, 2019; Lerdpornkulrat et al., 2019; Campbell and Kapp, 2020).

Regarding the use of questionnaires, scales or self-reports, we found several works that used participants' digital literacy histories as instruments. Through them, the researcher could learn first-hand about the sample's personal experience of digital literacy, the previous knowledge they possess, the digital skills they had mastered, those they lack, or those they consider they should improve. It also included the sample's vision regarding the use and employment of digital resources in teaching practice (Kajee and Balfour, 2011; Robertson et al., 2012; Pequeño et al., 2017; Elliott et al., 2018).

In the case of scales, we found two papers that employed a Likert-scale elaborated *ad hoc*. We also found studies that employed standardized scales like the Information Literacy Assessment Scale for Education (ILAS-ED), the Digital Literacy Scale, or the E-Learning Attitudes Scale.

Some of the studies we reviewed used semi-structured interviews as a means of monitoring and providing feedback to the students Table 3; (Kajee and Balfour, 2011; Alfonzo and Batson, 2014; Gill et al., 2015; Carl and Strydom, 2017; Elliott et al., 2018; Elphick, 2018; Ata and Yildirim, 2019; Campbell and Kapp, 2020).

**Table 3:** Assessment intervention in the reviewed studies.

**Research**	**Timetable for the implementation of each instrument**	**Direct comments**	**Task-specific performance**	**Overall task performance**
Alfonzo and Batson (2014)	Pre-evaluation, post-evaluation and follow-up evaluation using Qual-trics software	Comparison and improvement of the results obtained through the Qual-trics software	Learning the ZOTERO platform at the end of the invention	Mastery of digital bibliographic research and ZOTERO
Ata and Yildirim (2019)	During the intervention	Does not specify	Does not specify	Carecen of digital skills to find, evaluate, create, and communicate
Ball (2019)	During the intervention	Tests throughout the development of the subject through portfolios	Feedback of the results of the questionnaires at the end of each module that showed improvements	Progressive mastery of digital skills
Botturi (2019)	Before and after the intervention	Agree with the participants on the contents and the evaluation	Yields are analyzed practice and evolution	Limited space in the curriculum
Campbell and Kapp (2020)	Before and after the intervention	Learning models and tasks to apply in the classroom	Inclusion of digital competences in curriculum design and monitoring of their development	Differences between resources in cemters and in households
Carl and Strydom (2017)	Before and after the intervention	Assessment through direct observation and class visits	Digital learning as part of teacher training	Digital writing support required
Domingo-Coscolla et al. (2020)	Before and after the intervention	Documentary analysis. Discussion groups and finally questionnaires	Digital literacy and content creation	Not all aspects of CDD are measured
Elliott et al. (2018)	Before and after the intervention	Through the delivery of weekly activities	Increased capacity to identify, select and apply digital reading	Not all students developed these skills
Elphick (2018)	Before and after the intervention	Performance is measured through direct observation and scales	Increasing the dominance of digital competence with iPads	A single discipline with a smaller number of staff and students
Gabriele et al. (2019)	Before and after the intervention	feedback on your programming experience and skills from questionnaires	Medium-high level of CT skills, combining design and programming skills	It must be applied in educational practice and not only at the laboratory level
Gill et al. (2015)	Before and after the intervention	3 stages of ict teaching capacity development in which each phase is evaluated	Practice itself as a learning tool	Minimal development where there is no real use of ICT for learning and teaching
Hamutoglu et al. (2019)	Before and after the intervention	Before and after the introduction by standardized instruments	Increased attitudes and skills	Only through EDMODO
Istenic et al. (2016)	Before and after the intervention	Describes the statement design framework and evaluation criteria for solving mathematical and digital problems	Their conceptions changed during the course of passive recipients to active producers of media content.	Control group without intervention
Kajee and Balfour (2011)	Before and after the intervention	Evaluates the results by semesters from accounts or observations	Increasing digital capacity	Large differences in terms of resources
Kuhn (2017)	Before and after the intervention	Evaluate performance through student presentations	Improving your digital skills and abilities	Scarcity of digital tools
Lerdpornkulrat et al. (2019)	Before and after the intervention	Formative assessment and feedback	Increased ability to search, evaluate, process and communicate information	Only the students of the experimental group participated in a formalized activity in the classroom
Paige et al. (2016)	Before and after the intervention	Development of conceptual and semiotic understandings.	Increasing digital literacy in content creation	It is only done with one app
Pequeño et al. (2017)	Before and after the intervention	Narrative research with digital ethnography,	Technological and social mediation	Focused solely on one degree
Robertson et al. (2012)	Before, during, and after the intervention	Throughout the process, personal reflections on their own experience are requested.	New understanding of literacy, particularly when digital stories are shared as part of the adult classroom experience	Only uses digital stories to gather information from the sample
Sharp (2018)	Before and after the intervention	Performance is evaluated after each practice	Increased perceived levels of confidence and importance of digital literacy	Does not indicate assessment instruments
Tomczyk et al. (2020)	Before and after the intervention	Reflections and own experiences on e-leawrning at the end of each course	Increasing digital competence	Does not indicate assessment instruments
Vinokurova et al. (2021)	Before, during, and after the intervention	Observation, analysis and pedagogical design and surveys during the intervention	Increasing professional skills, information culture and digital literacy	Insufficient digital resources

As for the sequence through which the different interventions were developed, we found two types—first, those that divided the contents in time, as is the case of the work of Kajee and Balfour (2011), who covered a first semester digital writing from online classes, self-instructions and face-to-face classes in a specific laboratory, and in a second semester was exposed to different digital research techniques, following the same methodology. In contrast, we spotted the second type, where the same technique was followed throughout the study, as is the case of Robertson et al. (2012). They applied digital stories as a tool for the development of the activity, but also the evaluation of the competency. In the research carried out by Lerdpornkulrat et al. (2019), it is apparent that with the use of the rubric, the teacher gave them an example of the work and asked them all to practice evaluating and grading this work. In this way, they could check if they understood how to use a rubric. They then used the rubric to self-assess their work. After receiving feedback, both groups of students revised and resubmitted their completed projects again.

In the investigation by Elliott et al. (2018), the intervention was structured in work modules with the following sequence of sessions: they were introduced in the first session with opportunities for group discussions and questions. Essential module reading was provided in weekly online study units and module workshops integrated academic reading and writing activities, such as paraphrasing and referencing, with module content.

In the study by Ball (2019), in the first year, the students took modules on publishing history, culture, markets, and media. In the second year, the intervention was based on their publishing skills, reading for writing development, and grammar and general literacy.

Hamutoglu et al. (2019) organized their intervention in different weeks, such that during the first week of the 14-week semester, the instructor oriented the students for the course and administered pre-tests. In the following week, students were provided with a session on the Edmodo platform and orientation training on the course content.

In the work of Gabriele et al. (2019), the experimental research plan (i.e., activities to be performed, methodology to be adopted) was established over 4 months followed by the organization of the reading material (power point presentations, introductory videos of the software, handouts, *ad hoc* created applications as examples).

We also found interventions that had very short time durations, but provide daily detail of the contents and interventions. Similarly, Alfonzo and Batson (2014) dedicate 1 day to the search and orientation in digital resources, 1 day to the APA standards, and 3 days to develop and use a specific application.

In the research by Istenic et al. (2016), the intervention was based on six different types of tasks related to a variety of mathematical problems, including problems with redundant data, problems with multiple solutions, problems with multiple paths to the solution, problems with no solution, mathematical problems in logic, and problems with insufficient information.

In some interventions, the sequence through which they are developed is the very development of the subject of the degree course from which they are implemented, as is the case of the work of Gill et al. (2015).

In the work of Carl and Strydom (2017), students were first familiarized with the devices and then introduced to electronic portfolios, which helped them to create blogs that serve as platforms for electronic portfolios, and guided them on how to collect artifacts and how to reflect and share content.

In one work we found narrative was used as a technique so that the students could later present their work, analyze it in groups, rework it and present it again to their classmates. Kuhn (2017), Pequeño et al. (2017), and Elphick (2018) followed this model.

Adopting a novel consultative approach, Botturi (2019) co-designed the intervention with his students in two steps: they were surveyed 4 weeks before the start of the course and asked to choose between two options: an overview of different topics/methods/experiences, or an in-depth exploration of one or two topics/methods/experiences. All respondents indicated a preference for the first option and provided indications of the topics they wished to cover (see Tables 4, 5).

**Table 4:** Assessment instruments used in the instructional intervention in the reviewed studies.

**Research**	**Questionnaires-self-reports-rating scales-semantic differential**	**Wallet physical/virtual**	**Interviews-Reports**	**Evaluation of the effects of the intervention**	**Satisfaction**	**Comments-Individual-Group**
Alfonzo and Batson (2014)	Information literacy assessment scale for education (ILAS-ED)	Observations on student work	Does not specify	Post-evaluation of the competencies from the qualtrics software	Learning and satisfaction for participating students	Significant effects on previous methods of instruction
Ata and Yildirim (2019)	Digital literacy scale	Does not specify	Does not specify	The final evaluation confirms the mastery of digital competences	Attitudinal, cognitive and are predictors of digital literacy	Domain alto and positive perceptions of digital literacy
Ball (2019)	Article editing of at least 1,500 words of additional content to the article–500–word report detailing the choice of edits made and the approach used	Edited portfolio	Weekly blog through Pebblepad (an electronic portfolio platform), detailing and explaining the work done that week	1,090 edits in 124 articles, creating six new articles	High capacity for digital editing and publication of content	Mastery and monitoring of competencies after the training course
Botturi (2019)	*Ad hoc* elaborate Likert scale	Does not specify	Follow-up interviews	Greater digital self-efficacy	Critical assessment of obstacles to implementing DML	Ability to integrate DML
Campbell and Kapp (2020)	Questionnaires that provide background on participants' biographies, perceptions, and experiences with technology	Reflections - justification of their use of technology - narratives of the difficulties experienced	Video recording, semi-structured - focus group interview	Increasing understanding of digital learning possibilities	Complementary tool and means to participate and not as an intentional remedy	Digital non-competition is a barrier today
Carl and Strydom (2017)	*Ad hoc* elaborate Likert scale	Individual and virtual	Recorded interviews: reflection, training, professional development, and social dimensions of the e-portfolio	Integration of electronic portfolios as tools for reflection	High institutional expectations	Digital growth and development through the use of digital portfolios
Domingo-Coscolla et al. (2020)	*Ad hoc* elaborate Likert scale	Does not specify	Focus groups	Promoting digital literacy and digital content creation	Insufficient C DD proficiency	Three institutional actions on CDD to be considered in university curricula
Elliott et al. (2018)	Essay of 3,000 words on the theories of learning—group oral presentation	Portfolio of 3,000 words. The portfolio was divided into three sections that required students to relate different phases of their personal education experiences to theory.	Semi-structured questionnaires, mainly quantitative, at the beginning and end of the academic year	Difficulties as part of the process	Students' expectations of achievement as the course progressed	Scaffolding strategies with a positive effect on digital self-efficacy
Elphick (2018)	Free text surveys—*ad hoc* elaborate Likert scale	Does not specify	Semi-structured interview with small groups	Correlations between classrooms rich in technology and digital self-efficacy	The use of iPads has a positive impact on digital behaviors and perceptions about digital skills	Digital competence as a key skill in teachers
Gabriele et al. (2019)	*Ad hoc* elaborate Likert scale	Does not specify	Tests to check the level of abstraction, parallelism, logistics, synchronization, and control	practical applicability of the intervention	Elaboration of digital material from games with Scratch Software	Increased knowledge and digital skills
Gill et al. (2015)	Interviews developed in 6 phases	Does not specify	Interviews developed in 6 phases	development is proportionate to opportunities to observe and/or use ICT for learning	Classroom experience enables and accelerates the development of digital literacy	The development of digital literacy as a key challenge for future donors
Hamutoglu et al. (2019)	E-Learning attitudes scale—digital literacy scale	Does not specify	Does not specify	Relevant results in terms of avoidance	The trend is one of the most significant predictors of digital literacy skills.	Effectiveness of treatment on participants' attitudes toward e-learning platforms
Istenic et al. (2016)	Performance analysis—analysis of written reflections—pre- and post-test scores-reflections of the participants	Does not specify	Does not specify	Increases in digital pedagogical competences	Instructional approach with digital storytelling and multi-mode design to facilitate learning	Transfer of ICT competencies and their integration into teaching
Kajee and Balfour (2011)	Digital literacy stories of the participants (collected at the beginning of the semester)	Remarks of student work—access and sufficiency	Semi-structured interviews	Digital practice as valuable and social knowledge	Influence of the social context	Digital literacy as a contribution and influence to learning
		surveys—journal of researcher's reflections				
Kuhn (2017)	*Ad hoc* elaborate likert scale	Does not specify	Focus groups	Obtaining new literacies from digital practice	Need for support and guidance in these contents	Redesign of the PLE of the students.
Lerdpornkulrat et al. (2019)	Questionnaires developed *ad hoc*—standardized questionnaires	Rubric	Does not specify	Developing self-efficacy related to digital literacy	Increase in self-efficacy in information literacy	The rubric as an appropriate tool to measure learning outcomes related to information literacy
Paige et al. (2016)	*Ad hoc* elaborate Likert scale	Does not specify	Does not specify	experiences and reflections of the PST on Slowmation as an educational tool	Modeling of best practice evaluation tools.	Digital literacy skills development
Pequeño et al. (2017)	Transmedia narratives	Does not specify	Comments and recommendations made in the group work	Transmedia education as a process of technological mediation and social	Digital skills that students incorporate into internships design, analysis, production, and dissemination of transmedia content	Creation and dissemination of transmedia content
Robertson et al. (2012)	Personal digital story	Remarks of student work—journal of researcher's reflections	Does not specify	Digital stories as an appropriate tool for evaluation and reflection	Multi-literacy	Evidence of transformative pedagogy
Sharp (2018)	*Ad hoc* elaborate likert scale	Does not specify	Does not specify	Increasing prevalence of digital learning environments.	Greater involvement in digital practices	Collaborative digital literacy practices
Tomczyk et al. (2020)	*Ad hoc* elaborate likert scale	Does not specify	Does not specify	Need for more training	Need for more studies to identify digital gaps	Achievement Learning Autonomy Adaptation
Vinokurova et al. (2021)	Does not specify	Does not specify	Does not specify	Educational paradigm shift in terms of the content of education	Digital transformation	Increased opportunities for teachers to offer and disseminate ICTs if they have good digital literacy

**Table 5:** Treatment fidelity.

**Research**	**Pertinence**	**Meetings**	**Feedback**	**Reliability and validity assessment**	**Maintenance and generalization**	**Other controls**	**Feedback**
Alfonzo and Batson (2014)	Horizontal relevance	Does not specify	Feedback to the student at the end of the course	Does not specify	Pre-post-follow-up evaluation	Agreement between observers collecting data	The duration of the workshops is short
Ata and Yildirim (2019)	Horizontal relevance	Does not specify	Feedback to students after the completion of each phase	Reliability Validity	Pre-post-intervention evaluation	A single researcher	Does not indicate the process or sessions
Ball (2019)	Horizontal relevance	Does not specify	Feedback to students after each module	Consistency	Pre-post-intervention evaluation	A single researcher	Does not use standardized instruments
Botturi (2019)	Horizontal relevance	Does not specify	Continuous feedback to students on each task	Consistency	Pre-post-intervention evaluation	A single researcher	Does not use records such as interviews or portfolios
Campbell and Kapp (2020)	Horizontal relevance	Does not specify	Feedback at the end of the intervention	Does not specify	Pre-post-intervention evaluation	A single researcher	Does not indicate the process or sessions
Carl and Strydom (2017)	Horizontal relevance	Does not specify	Feedback to students at the end of the course	Does not specify	Pre-post-intervention evaluation	A single researcher	Does not specify the duration
Domingo-Coscolla et al. (2020)	Horizontal relevance	Does not specify	Feedback to students at the end of the intervention	Reliability Validity	Pre-post-intervention evaluation	Agreement between observers collecting data	Does not use records such as interviews or portfolios
Elliott et al. (2018)	Horizontal relevance	Does not specify	Feedback to students after each session	Reliability Validity Consistency Exploratory factor analysis	Pre-post-intervention evaluation	Agreement between observers collecting data	Does not use standardized instruments
Elphick (2018)	Horizontal relevance	Does not specify	Feedback to students after each session	Consistency	Pre-post-intervention evaluation	A single researcher	Does not use standardized instruments
Gabriele et al. (2019)	Horizontal relevance	Does not specify	feedback on your programming experience and skills from questionnaires	Reliability Consistency Validity	Pre-post-intervention evaluation	Does not specify	Does not use records such as interviews or portfolios
Gill et al. (2015)	Horizontal relevance	Does not specify	Feedback to students in each subject	Reliability Consistency Validity Exploratory factor analysis	Pre-post-follow-up evaluation	Do not specify	Does not apply any self-assessment scale
Hamutoglu et al. (2019)	Horizontal relevance	Does not specify	Feedback to students with the scores of each standardized instrument	Reliability Validity	Pre-post-intervention evaluation	A single researcher	Does not use records such as interviews or portfolios
Istenic et al. (2016)	Horizontal relevance	Does not specify	Feedback to students after completing each task (6)	Reliability Validity	Pre-post-intervention evaluation	Do not specify	Does not apply any self-assessment scale
Kajee and Balfour (2011)	Horizontal relevance	Does not specify	Student feedback at the end of each semester	Does not specify	Pre-post-intervention evaluation	A single researcher	Only applicable within the university and within the laboratory itself
Kuhn (2017)	Horizontal relevance	Does not specify	Continuous feedback after each student presentation	Vaqlidez	Pre-post-follow-up evaluation	Do not specify	Does not use standardized instruments
Lerdpornkulrat et al. (2019)	Horizontal relevance	Does not specify	Feedback from the researcher and self-assessment	Reliability Consistency Validity Exploratory factor analysis	Pre-post-intervention evaluation	A single researcher	Does not use records such as interviews or portfolios
Paige et al. (2016)	Horizontal relevance	Does not specify	Feedback after the intervention	Validity	Pre-post-intervention evaluation	Do not specify	Does not specify the duration
Pequeño et al. (2017)	Horizontal relevance	Does not specify	Feedback after the intervention	Consistency Validity	Pre-post-intervention evaluation	Do not specify	Does not use standardized instruments
Robertson et al. (2012)	Horizontal relevance	Does not specify	Continuous feedback from their own experiences	Does not specify	Pre-post-follow-up evaluation	Agreement between observers collecting data	Does not apply any self-assessment scale
Sharp (2018)	Horizontal relevance	Does not specify	Feedback after the intervention	Consistency Exploratory factor analysis	Pre-post-intervention evaluation	Do not specify	Does not use standardized instruments
Tomczyk et al. (2020)	Horizontal relevance	Does not specify	Feedback after the intervention	Reliability Consistency Validity Exploratory factor analysis	Pre-post-intervention evaluation	Do not specify	Does not use records such as interviews or portfolios
Vinokurova et al. (2021)	Horizontal relevance	Does not specify	Feedback from students through their own experience	Validity	Pre-post-follow-up evaluation	Do not specify	Does not indicate the process or sessions

Indicators and controls used in the instructional intervention in the empirical studies reviewed II.

The limitations of our search are listed in Table 6. At the theoretical level, we encountered studies that were not very current, missing research questions or hypotheses, or even missing objectives. At the statistical level, we found several studies had a small or unrepresentative sample.

**Table 6:** Limitations of the instructional interventions described in the empirical studies reviewed.

**Research**	**Background limitations**	**Limitations on participants**	**Limitations of the instrument**	**Program limitations**	**Limitations of results**	**Discussion on limitations and conclusions**	**General limitations**	**Comments**
Alfonzo and Batson (2014)	The research question is missing Missing assumptions or forecasts Missing targets	Reduced sample Non-representative sample	Non-validity and reliability of instruments with their own data	Non-grouping	No graphs or tables They do not analyze each variable Not analyzing generalization effects	Does not indicate reliability and validity assessment	No ethical controls (informed acceptance to participate, confidentiality...)	Sample must be larger
Ata and Yildirim (2019)	The research question is missing	Lack of inclusion and exclusion criteria	No tasks Do not record the entire process	Non-grouping	They do not analyze each variable	They do not compare with previous current studies	No ethical controls (informed acceptance to participate, confidentiality...)	Few evaluation strategies
Ball (2019)	The research question is missing Missing assumptions or forecasts	No method	Non-validity and reliability of instruments with their own data Instruments unknown and not provided for in the Annex	Non-grouping	No graphs or tables They do not analyze each variable	They do not compare with previous current studies	No ethical controls (informed acceptance to participate, confidentiality...)	Does not indicate the sample
Botturi (2019)	The research question is missing	Reduced sample Non-representative sample	Non-validity and reliability of instruments with their own data Instruments unknown and not provided for in the Annex	Non-grouping	They do not analyze each variable	They do not compare with previous current studies	No ethical controls (informed acceptance to participate, confidentiality...)	Few evaluation strategies
Campbell and Kapp (2020)	The research question is missing Missing assumptions or forecasts	Lack of inclusion and exclusion criteria	No tasks Do not record the entire process	Non-grouping	They do not analyze each variable	Does not indicate reliability and validity assessment current previews	No ethical controls (informed acceptance to participate, confidentiality...)	Sample must be larger
Carl and Strydom (2017)	The research question is missing Missing assumptions or forecasts Missing targets	Reduced sample Non-representative sample	Non-validity and reliability of instruments with their own data Instruments unknown and not provided for in the Annex	Non-grouping No duration	No graphs or tables They do not analyze each variable Not analyzing generalization effects	Does not indicate reliability and validity assessment	No ethical controls (informed acceptance to participate, confidentiality...)	Sample must be larger
Domingo-Coscolla et al. (2020)	The research question is missing Missing assumptions or forecasts	Reduced sample Non-representative sample	Non-validity and reliability of instruments with their own data Instruments unknown and not provided for in the Annex	Non-grouping	They do not analyze each variable	They do not compare with previous current studies	No ethical controls (informed acceptance to participate, confidentiality...)	Sample must be larger
Elliott et al. (2018)	The research question is missing Missing assumptions or forecasts	Lack of inclusion and exclusion criteria Reduced sample Non-representative sample	Non-validity and reliability of instruments with their own data Instruments unknown and not provided for in the Annex	Non-grouping	No graphs or tables They do not analyze each variable	They do not compare with previous current studies	No ethical controls (informed acceptance to participate, confidentiality...)	Sample must be larger
Elphick (2018)	The research question is missing Missing assumptions or forecasts	Lack of inclusion and exclusion criteria	Non-validity and reliability of instruments with their own data Instruments unknown and not provided for in the Annex	No number of sessions	They do not analyze each variable	They do not compare with previous current studies	No ethical controls (informed acceptance to participate, confidentiality...)	The application of standardized chords and instruments is lacking. Few evaluation strategies
Gabriele et al. (2019)	Obsolete fonts	Reduced sample Non-representative sample	Non-validity and reliability of instruments with their own data Instruments unknown and not provided for in the Annex	Non-grouping	Only the publication is compared	They do not compare with previous current studies	No ethical controls (informed acceptance to participate, confidentiality...)	Sample must be larger
Gill et al. (2015)	The research question is missing Missing assumptions or forecasts Missing targets	Reduced sample Non-representative sample	Non-validity and reliability of instruments with their own data	Non-grouping	No graphs or tables They do not analyze each variable Not analyzing generalization effects	They do not compare with previous current studies	No ethical controls (informed acceptance to participate, confidentiality...)	Sample must be larger
Hamutoglu et al. (2019)	The research question is missing	Lack of inclusion and exclusion criteria	No tasks	Non-grouping	Only the publication is compared	The answer to the research question is not indicated	No ethical controls (informed acceptance to participate, confidentiality...)	Few evaluation strategies
Istenic et al. (2016)	The research question is missing Missing assumptions or forecasts Missing targets	Lack of inclusion and exclusion criteria	Non-validity and reliability of instruments with their own data Instruments unknown and not provided for in the Annex	Non-grouping	No graphs or tables They do not analyze each variable Not analyzing generalization effects	They do not compare with previous current studies	No ethical controls (informed acceptance to participate, confidentiality...)	The application of standardized chords and instruments is lacking. Few evaluation strategies
Kajee and Balfour (2011)	Obsolete fonts The research question is missing Missing assumptions or forecasts	Reduced sample Non-representative sample	Non-validity and reliability of instruments with their own data Instruments unknown and not provided for in the Annex	Not who implemented	No graphs or tables They do not analyze each variable Not analyzing generalization effects	Does not indicate Reliability and Validity Assessment	Key information to replicate the intervention is missing	Sample must be larger
Kuhn (2017)	The research question is missing Missing assumptions or forecasts Missing targets	Reduced sample Non-representative sample	Non-validity and reliability of instruments with their own data Instruments unknown and not provided for in the Annex	No number of sessions Not who implemented	No graphs or tables They do not analyze each variable	They do not compare with previous current studies	No ethical controls (informed acceptance to participate, confidentiality...)	Sample must be larger
Lerdpornkulrat et al. (2019)	Missing assumptions or forecasts	Lack of inclusion and exclusion criteria	No tasks Do not record the entire process	Does not indicate instruction procedure	No practical and theoretical applications	No explicit limitations	No ethical controls (informed acceptance to participate, confidentiality...)	Does not use the wallet
Paige et al. (2016)	The research question is missing Missing assumptions or forecasts Missing targets	Reduced sample Non-representative sample	Non-validity and reliability of instruments with their own data Instruments unknown and not provided for in the Annex	Non-grouping	No graphs or tables They do not analyze each variable Not analyzing generalization effects	They do not compare with previous current studies	No ethical controls (informed acceptance to participate, confidentiality...)	Sample must be larger
Pequeño et al. (2017)	The research question is missing Missing assumptions or forecasts Missing targets	Reduced sample Non-representative sample	Non-validity and reliability of instruments with their own data Instruments unknown and not provided for in the Annex	No number of sessions Not who implemented	No graphs or tables They do not analyze each variable	They do not compare with previous current studies	No ethical controls (informed acceptance to participate, confidentiality...)	Sample must be larger
Robertson et al. (2012)	Obsolete fonts The research question is missing Missing assumptions or forecasts Missing targets	Reduced sample Non-representative sample	Non-validity and reliability of instruments with their own data Inadequacy of the age course of the instruments Instruments unknown and not provided for in the Annex	Not who implemented	No graphs or tables They do not analyze each variable Not analyzing generalization effects	Does not indicate Reliability and Validity Assessment	It's not an experimental intervention study, it's just a pre-post group Key information to replicate the intervention is missing No ethical controls (informed acceptance to participate, confidentiality...)	The application of standardized chords and instruments is lacking. Few evaluation strategies
Sharp (2018)	The research question is missing Missing assumptions or forecasts	Lack of inclusion and exclusion criteria	Non-validity and reliability of instruments with their own data Instruments unknown and not provided for in the Annex	No number of sessions Not who implemented	No graphs or tables They do not analyze each variable	They do not compare with previous current studies	Key information to replicate the intervention is missing	The application of standardized chords and instruments is lacking. Few evaluation strategies
Tomczyk et al. (2020)	Missing research question Missing assumptions or forecasts	Lack of inclusion and exclusion criteria	No tasks Do not record the entire process	Non-grouping	They do not analyze each variable	They do not compare with previous current studies	No ethical controls (informed acceptance to participate, confidentiality...)	Few evaluation strategies
Vinokurova et al. (2021)	The research question is missing Missing assumptions or forecasts Missing targets	Lack of inclusion and exclusion criteria	No tasks Do not record the entire process	Non-grouping	They do not analyze each variable	They do not compare with previous current studies	No ethical controls (informed acceptance to participate, confidentiality...)	Does not indicate the procedure or the participants or the sessions

Analyzing the interventions themselves, we identified a few limitations, especially in those studies that neither indicates the tasks, record the entire process, or lack key information to replicate the intervention. In some studies, key information relating to the person carrying out the intervention was missing, particularly on whether they had the specific training for this purpose. Another limitation that was identified was that very few evaluation strategies were in place to evaluate the interventions (see Table 7).

**Table 7:** Treatment fidelity.

**Research**	**Moment**	**Comparison of the control group**	**Sequence of instruction**	**Previous written protocol**	**Comparable instructor training**	**File**	**Uniform and standard application**
Alfonzo and Batson (2014)	Pre During Expose Follow	Evaluate the group in general	3 workshops: Library Orientation, APA style, ZOTERO	Day 1: Library orientation, APA style. Day 2, 3, and 4: ZOTERO	Does not specify	Pre-evaluation, post-evaluation, and follow-up evaluation using qualtrics software	Equal application of the program to all students: same duration, sequence, tasks, and context
Ata and Yildirim (2019)	During Expose Follow	Evaluate the group in general	Does not specify	Does not specify	Does not specify	Does not specify	Equal application of the program to all students: same duration, sequence, tasks, and context
Ball (2019)	During Expose Follow	Evaluate the group in general	Modules of history and editorial culture, markets, and media. Editorial Skills Module, Reading for Writing, and Grammar Development and General Literacy	Does not specify	Does not specify	Portfolios and weekly blog	Equal application of the program to all students: same duration, sequence, tasks, and context
Botturi (2019)	During Expose Follow	Evaluate the group in general	Agreed with students that provided instructions on the topics they wished to cover	Does not specify	Does not specify	Balance	Equal application of the program to all students: same duration, sequence, tasks, and context
Campbell and Kapp (2020)	During Expose Follow	Evaluate the group in general	Does not specify	Does not specify	Does not specify	Questionnaires, portfolio, and interviews	Equal application of the program to all students: same duration, sequence, tasks, and context
Carl and Strydom (2017)	Pre During Expose Follow	They evaluate the group in general although I am divided into two subgroups	Stages: familiarization, indexing, graphing and cartography, and interpretation	Familiarization -blo-share	Does not specify	-Recorded interviews - portfolio	Equal application of the program to all students: same duration, sequence, tasks, and context
Domingo-Coscolla et al. (2020)	During Expose Follow	Evaluate the group in general	Does not specify	Does not specify	Does not specify	Scales and focus groups	Equal application of the program to all students: same duration, sequence, tasks, and context
Elliott et al. (2018)	During Expose Follow	Evaluate the group in general	Sessions with opportunities for group discussions and questions. Module essential reading was provided in weekly online study units	Does not specify	Broader university support from support staff specializing in academic skills in the “learning development team” and library staff.	Questionnaires, essays, and portfolio	Equal application of the program to all students: same duration, sequence, tasks, and context
Elphick (2018)	During Expose Follow	Evaluate the group in general	Conferences and seminars—direct observation—scales—interviews	Does not specify	Training sessions facilitated by an Apple professional Authorized Development Coach	Narratives—presentations—classroom observations—comments and feedback—audiovisual recordings	Equal application of the program to all students: same duration, sequence, tasks, and context
Gabriele et al. (2019)	During Expose Follow	Evaluate the group in general	1. Experimental research plan 2. The reading material was organized (power point presentations, introductory videos of the software, brochures, applications created *ad hoc* as examples)	Does not specify	Does not specify	Scales and individual tests	Equal application of the program to all students: same duration, sequence, tasks, and context
Gill et al. (2015)	Pre During Expose Follow	Evaluate the group in general	Of the different subjects related to ICT in the career	Of the different subjects related to ICT in the career	Does not specify	Interviews	Equal application of the program to all students: same duration, sequence, context tasks
Hamutoglu et al. (2019)	During Expose Follow	Evaluate the group in general	Preliminary tests of the first week. In the following week session on the Edmodo platform and an orientation training on the content of the course	Does not specify	Does not specify	Two standardized scales	Equal application of the program to all students: same duration, sequence, context tasks
Istenic et al. (2016)	Pre During Expose Follow	Evaluate the group in general	Six tasks	Students completed the pre-test before the start of the study and the subsequent test 15 days later.	Does not specify	Digital Literacy Stories—Pre and Post-Assessment	Equal application of the program to all students: same duration, sequence, context tasks
Kajee and Balfour (2011)	Pre During Expose Follow	Evaluation of the intervention group and another equivalent control group to verify differential efficacy	Semester 1: Digital Writing Semester 2: Digital Research	Does not specify	Does not specify	Digital literacy stories—semi-structured interviews—observations—access and sufficiency surveys—journal of researchers' reflections	Equal application of the program to all students: same duration, sequence, context tasks
Kuhn (2017)	During Expose Follow	Evaluation of the intervention group and another equivalent control group to verify differential efficacy	Scales—exhibition—discussion groups	Does not specify	Does not specify	Narratives—exhibitions—classroom observations—comments and feedback—audiovisual recordings	Equal application of the program to all students: same duration, sequence, context tasks
Lerdpornkulrat et al. (2019)	During Expose Follow	Only the GC participates in a formalized face-to-face activity based on the use of the course rubric as a self-assessment tool	Through the rubric they were able to self-evaluate your own work After receiving feedback, both groups of students reviewed and resubmitted their feedback Complete projects again	Does not specify	Does not specify	Questionnaires developed *ad hoc*—standardized questionnaires	only the students of the experimental group participated in a formalized activity in the classroom
Paige et al. (2016)	Pre During Expose Follow	Evaluate the group in general	Slowmation, vivas, digital narratives, roundtables, interviews and oral assessments	Slow	Does not specify	Pre- and post- intervention test—Scale	Equal application of the program to all students: same duration, sequence, context tasks
Pequeño et al. (2017)	During Expose Follow	Evaluation of the intervention group and another equivalent control group to verify differential efficacy	Narrative—characteristics—exhibition—analysis—reworking—exhibition and possibilities	Digital ethnography for examine relations with technologies and the media and how they mediate in the configuration of subjectivities	Does not specify	Narratives—exhibitions—classroom observations—comments and feedback—audiovisual recordings	Equal application of the program to all students: same duration, sequence, context tasks
Robertson et al. (2012)	Pre During Expose Follow	Evaluate the group in general	Digital stories. After the presentation, you are asked to write a written reflection describing your experience	Content analysis and categorization	Does not specify	Digital literacy stories of the—observations—journal of researcher's reflections	Equal application of the program to all students: same duration, sequence, context tasks
Sharp (2018)	During Expose Follow	Evaluation of the intervention group and another equivalent control group to verify differential efficacy	Does not specify	Does not specify	Does not specify	Scales	Equal application of the program to all students: same duration, sequence, context tasks
Tomczyk et al. (2020)	During Expose Follow	Evaluate the group in general	Unspecified	Does not specify	Does not specify	Scale	Equal application of the program to all students: same duration, sequence, context tasks
Vinokurova et al. (2021)	During Expose Follow	Evaluate the group in general	Does not specify	Does not specify	Does not specify	Theoretical analysis of the pedagogical experience, interpretation of scientific data, pedagogical design method (planning, modeling, and conducting classes), and analysis of empirical data in the form of a survey	Equal application of the program to all students: same duration, sequence, context tasks

Indicators and controls used in the instructional intervention in the empirical studies reviewed.

Similarly, gaps were found regarding ethical controls, where in some studies the main limitation was that ethical controls were non-existent or not specified (Robertson et al., 2012; Istenic et al., 2016; Kuhn, 2017; Elphick, 2018; Ata and Yildirim, 2019; Tomczyk et al., 2020).

**Figure 3:**
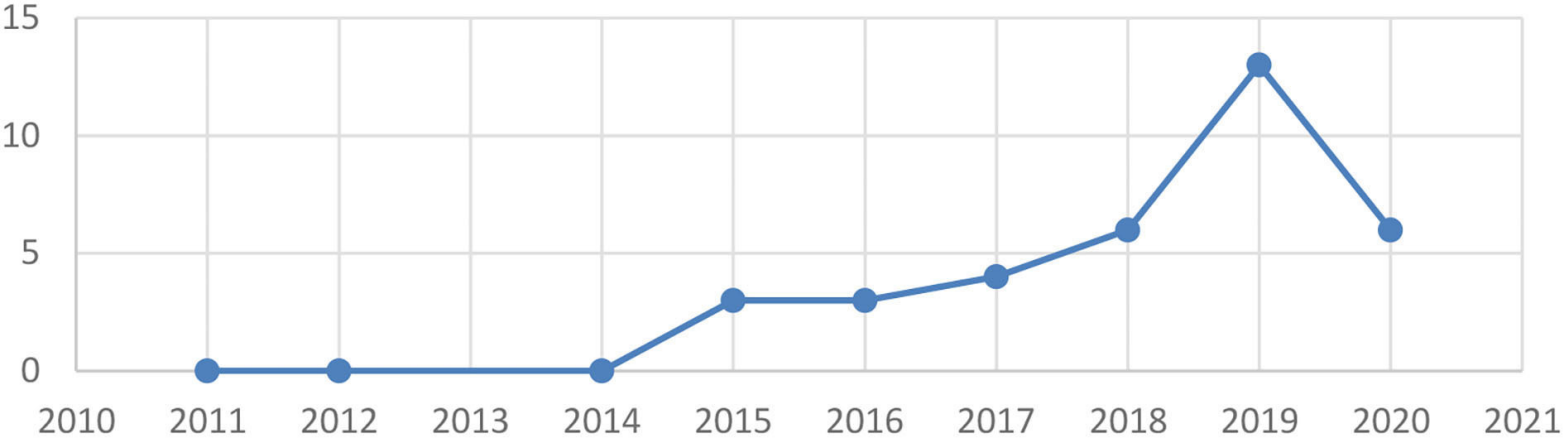
Evolution over years of the samples used in the studies from 2010 to 2021.

Figure 3 shows the evolution over the years of the samples used in each of the studies from 2011 to 2020.

**Figure 4:**
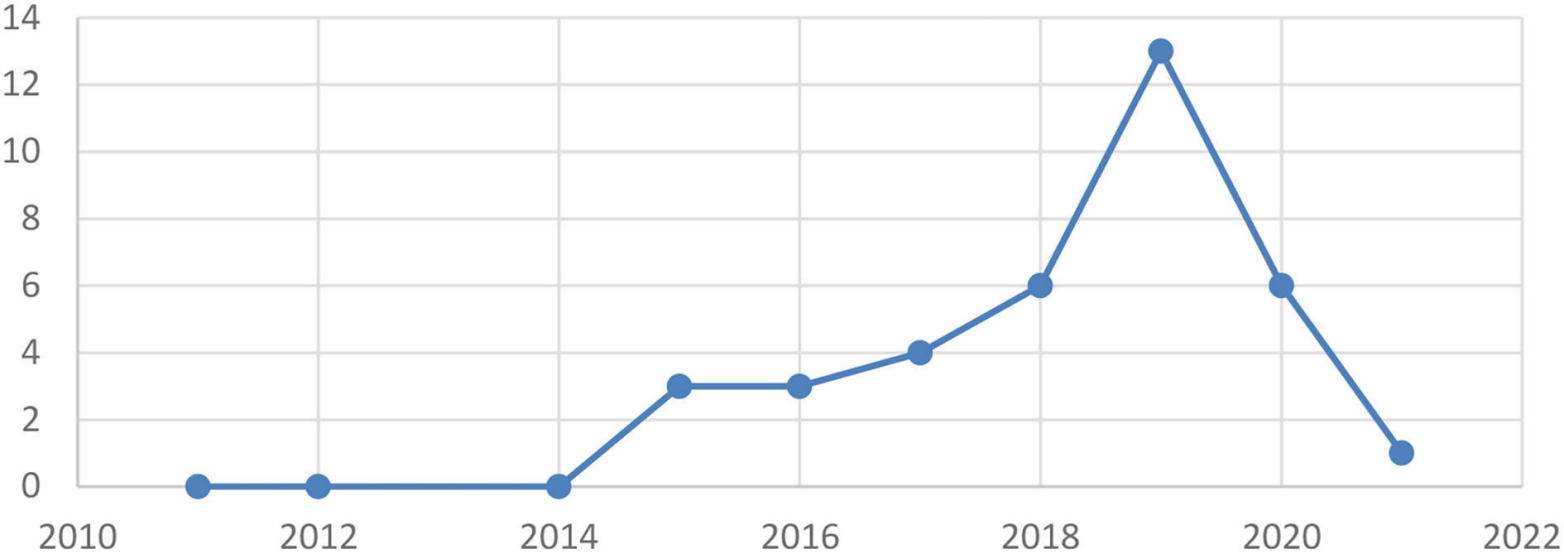
Evolution over years of the controls used in studies from 2010 to 2021.

Figure 4 shows the evolution over the years of the controls used in each of the studies from 2011 to 2021.

## Discussion

This work aimed to analyze the empirical evidence found in international studies between 2011 to 2021 related to the digital literacy of university students, including those pursuing degrees in education. This objective has been met.

Regarding the first focus related to literacy, this paper highlighted the fact that studies from the West are the most prevalent in this field (Çoklar et al., 2017; Ata and Yildirim, 2019; Hamutoglu et al., 2019; Sujarwo et al., 2022), which correspond to cross-sectional studies, mostly employing instruments such as “the Digital Literacy Scale” developed by Ng (2012), and “the information literacy self-efficacy scale (ILS)” developed by Kurbanoglu et al. (2006). Regarding the level of mastery, the results showed an upper intermediate level of competence in information and digital literacy, communication, and collaboration, but a low intermediate level in terms of digital content creation, particularly in the creation and dissemination of multimedia content using different tools (López-Meneses et al., 2020; Moreno et al., 2020).

Regarding the second focus, digital literacy in university students, this study reviewed the various contributions of other works and found the presence of a competent group in this field, which makes efficient use of both the Internet and digital media (Çoklar et al., 2016; Ata and Yildirim, 2019; Lim and Newby, 2021). However, differences were also found in this collective relating to gender, where women were more competent than men in digital literacy, information literacy, technological literacy, and communicative literacy (Hamutoglu et al., 2019; López-Meneses et al., 2020; Navarro, 2020). However, on the other hand, we lso found studies that revealed particular gender gaps where men showed a higher propensity for DL, while women outperform men in the overall digital literacy test (Ata and Yildirim, 2019). Ata and Yildirim (2019) also found differences in DL between students where university students studying science or mathematics-related majors had higher levels of digital literacy than students majoring in social sciences or psychology fields (Ata and Yildirim, 2019; Chow and Wong, 2020).

And as for the third focus, digital literacy in future teachers, we found a dual use of digital literacy, in its social and leisure aspect (searching or maintaining friendships through social networks, sharing digital content, downloading content, or playing online games), and in its academic aspect (searching in search engines, working through online documents, organizing or synthesizing information from different processors, using computer programs to make presentations, edit images or content, or create audiovisual content (López-Meneses et al., 2020).

The main contribution of this review lies in its comparison between pre/post-pandemic studies, which show a great increase in the use of technologies in the educational world (across the curriculum), and research work focused on measuring the competencies of these devices (Baber et al., 2022). These new investigations have not only followed the line of previous ones but focused on the measurement of digital literacy and its influence on it by variables such as the degree of origin, gender, age, or being a digital native or immigrant (Castañeda-Peña et al., 2015; Çoklar et al., 2016; Castañeda et al., 2018; Ata and Yildirim, 2019; Gür et al., 2019; Hamutoglu et al., 2019; Lerdpornkulrat et al., 2019; González et al., 2020; Navarro, 2020; De Sixte et al., 2021). But there has been an expansion of the topics and variables that are studied in conjunction with digital literacy, among which we find as a novelty, the study of psycho-educational variables such as academic motivation (Chow and Wong, 2020), self-efficacy and motivation (Lerdpornkulrat et al., 2019), effort expectations (Nikou and Aavakare, 2021), and self-concept as a student and as a teacher (Yeşilyurt et al., 2016). The importance attached to the educational field, the identification of different roles or behaviors within the concept of digital literacy that is delimited, or even the types of uses within the concept of digital literacy (López-Meneses et al., 2020; Moreno et al., 2020; Navarro, 2020; Lim and Newby, 2021) are new trends.

Therefore, we can affirm that in this study the research predictions are fulfilled, in that the results found show relevant differences from international studies pre-post pandemic; and by different cultural backgrounds (Spanish Latin, Portuguese, Finnish...), gender, and personal digital resources. In terms of applications for educational practice, these results do not indicate that university students are competent in terms of digital literacy, although they demonstrate some competencies like online information search, information evaluation, information processing, information communication, and dissemination skills (Çoklar et al., 2016; Lerdpornkulrat et al., 2019). Therefore, there is the risk of training an incomplete student body in digital competence. For complete and comprehensive digital literacy for university students, especially future teachers, there is an urgent need to invest in digital literacy programs. This will ensure that the comprehensive digital competence of students corresponds to the use and employment of the Internet and digital devices in their teaching tasks (Gisbert et al., 2016), and be a guarantee of their integration into teaching practice (Aslan and Zhu, 2016; Nikou and Aavakare, 2021).

As for the limitations of this work, they are closely related to the seven indicators for analyzing study quality and effectiveness (Acosta and Garza, 2011), which are: alignment of theory, findings, reliability and validity, descriptive details of participants, and the study, sample, and consistency of findings and conclusions with the data (Risko et al., 2008). Along with evidence-based indicators, and effect sizes of studies (Díaz and García, 2016; Canedo-García et al., 2017). So future lines of research or work, should take into account overcoming these limitations, and embrace them in the face of their development.

The number of studies found in the systematic review is comparable to what is usual in this type of study and even higher. For example, in the exemplary systematic review by Scott et al. (2018), they identified only 29 studies that met the quality criteria, reviewing 50 years of studies published in the US, and of these, only four were quantitative. In the study by Borgi et al. (2020), they only found ten studies that fit the criteria in a very good analysis. Other systematic reviews go along the same lines, and in the same journal and section *Frontiers in Psychology*. For example, Dickson and Schubert (2020) and Liu et al. (2022) found only six studies in a review of great interest; the study by Nguyen et al. (2021) identified 18 eligible articles; Shou et al. (2022) with 12 studies included; or Tarchi et al. (2021); Huang (2022) found seven studies for quantitative analysis and eight for indirect evidence; Coxen et al. (2021) with 21 articles included in the focal analyzes of the systematic review. The number of studies to be representative is not defined by the number but by the existence of such studies. In a systematic review, all studies are reviewed, thus the population of published studies that fit the indicated criteria. With these studies, it was possible to do an analysis of objective indicators in a general comparison between studies; assessing the instruments used; examining the characteristics of the interventions such as strategies, instructional procedure, and psychological variables considered; comparing the fidelity controls of the treatments, which guarantees their rigor and their application in the terms prescribed by the empirical validation of the interventions; and reviewing the limitations of the studies and their contributions by years. These contributions were based on objective data from the studies and have been represented in tables and figures. In addition, a qualitative analysis is provided that highlights the value of intervention studies in relation to digital competence, and the key psychological variables that have been used. It is true that the studies published since 2010 were used, and that there could have been more studies before, but considering the evolution of this type of focus in relation to digital competence and the psychological variables involved, it is evident that the most interesting thing is to consider the recent years which is when its need and use has been generalized throughout the population.

## Conclusions

In general, the results show that university students are digitally literate and make efficient use of both the Internet and digital media. In this sense, we found an intermediate or higher level in skills related to communication and collaboration, such as through different chat rooms, platforms, and communication applications. But an intermediate-low level in terms of digital content creation, especially in the creation and dissemination of multimedia content. So, this should be one of the future competencies to increase in this group. Although there are differences according to gender, age, or degree of origin.

We have to invest in comprehensive digital literacy programs for teachers in initial training, which appears implicit in the training plans of their official studies. Digital literacy needs to be a part of the official curriculum, and be developed rather quickly as a separate subject but in an interdisciplinary manner throughout their training. In this way, they become digitally literate people capable of creating and generating digital content and possessing the necessary competencies and skills to use and share such content.

We must also invest in assessing teachers' self-perception. Only by knowing their opinion, skills, and shortcomings, can digital training programs be designed. Digital literacy is a predictor of good digital use and a predictor of the good use and employment of digital devices and the Internet in the future when they would be teaching.

The findings of this study compel us to consider the following: first, we need to rethink the form and manner in which future teachers are capacitated in digital literacy, if we are doing it in the best way, or if on the contrary there are gaps that should be solved. Second, we should take into account the contributions of the results found and their consequences to formulate effective intervention designs and strategies to effectively capacitate pre-service teachers in digital literacy.

## Data availability statement

The raw data supporting the conclusions of this article will be made available by the authors, without undue reservation.

## Author contributions

J-NS-G, NG-Á, IM-R, JG-M, and SB-C: conceptualization, methodology, software, writing—review and editing, visualization, supervision, and validation. NG-A: formal analysis, investigation, and resources: UAL, ULE, USAL, IPC, data curation, writing—original draft preparation, and funding acquisition. J-NS-G and NG-A: project administration. All authors contributed to the article and approved the submitted version.

## Funding

The generalx operating funds of the universities have been used Universidad de León (Spain), Universidad de Almería (Spain), Universidad de Salamanca (Spain), Instituto Politécnico de Coimbra and NICSH (Portugal).

## Conflict of interest

The authors declare that the research was conducted in the absence of any commercial or financial relationships that could be construed as a potential conflict of interest.

## Publisher's note

All claims expressed in this article are solely those of the authors and do not necessarily represent those of their affiliated organizations, or those of the publisher, the editors and the reviewers. Any product that may be evaluated in this article, or claim that may be made by its manufacturer, is not guaranteed or endorsed by the publisher.
